# Supplementary material for: On-chip integrated process-programmable sub-10 nm thick molecular devices switching between photomultiplication and memristive behaviour
Source: Nat Commun. 2022 May 24;13:2875. doi: 10.1038/s41467-022-30498-y (PMC9130281; doi:10.1038/s41467-022-30498-y)
Supplement: Supplementary file 1 — Supplementary Information [file 41467_2022_30498_MOESM1_ESM.pdf]

Supplementary Information

On-Chip Integrated Process-Programmable Sub-10 nm Thick Molecular Devices Switching between Photomultiplication and Memristive Behaviour

Tianming Li[1,2,3], Martin Hantusch[4], Jiang Qu[1,2,3], Vineeth Kumar Bandari[1,2,3], Martin Knupfer[4], Feng Zhu[1,2,3,5]⋆, Oliver G. Schmidt[1,2,3,6]⋆

[1]Center for Materials, Architectures and Integration of Nanomembranes (MAIN), Chemnitz University of Technology, 09126 Chemnitz, Germany

[2]Material Systems for Nanoelectronics, Chemnitz University of Technology, 09107 Chemnitz, Germany

[3]Institute for Integrative Nanosciences, Leibniz IFW Dresden, 01069 Dresden, Germany

[4]Institute for Solid State Research, Leibniz IFW Dresden, 01069 Dresden, Germany

[5]State Key Laboratory of Polymer Physics and Chemistry, Changchun Institute of Applied Chemistry, Chinese Academy of Sciences, 130022 Changchun, China

[6]School of Science, Dresden University of Technology, 01069 Dresden, Germany

⋆e-mail: zhufeng@ciac.ac.cn (F.Zhu); oliver.schmidt@main.tu-chemnitz.de (O.G.Schmidt)

The PDF file includes:

Supplementary Notes 1-13;

Supplementary Figures 1-17;

Supplementary Tables 1-3;

Supplementary References.

Supplementary Note 1. Comparison of different techniques for creating sub-10 nm thick molecular devices

A literature study is conducted to compare the rolled-up soft contact technology with other fabrication techniques for creating sub-10 nm thick molecular devices, as shown in Supplementary Table 1. Each technique contributes greatly to the development of molecular electronics[1, 2].

Bending induced by releasing built-in strain gradients in thin film layer stacks is an effective approach to self-assemble 2D planar nanomembranes into 3D micro- and nano-tubular architectures, which has provided a smart platform for various fundamental investigations as well as promising applications in the field of the micro- and nanosciences, including magnetics[3], biology[4], optics[5], robotics[6], and energy storage[7]. At the same time, this self-curling effect can also create a damage-free and self-adjusted top electrode on molecular layers (including self-assembled monolayers, deposited ultrathin molecular layers, and so on) after rolling, hence extending this technique to molecular electronics[8]. More importantly, the self-rolling process is compatible to conventional photolithography techniques. Therefore, fully integrated sub-10 nm thick molecular devices on wafer scale can be realized by rolled-up soft contacts. Based on this technique some significant achievements have been obtained.

The capability of rolled-up soft contacts is comparable to existing well-established fabrication techniques for creating sub-10 nm thick molecular devices on large scales such as nanoskiving[9], surface-diffusion-mediated deposition[10], pore-assisted carbon paint[11], and so on. Still, there are challenges to tackle when using the rolled-up nanotechnology. First, due to the ultrathin feature of the sacrificial and strained layers, any defect can lead to an uneven stress distribution, resulting in the failure of rolling. Second, the roughness of the top tubular and bottom finger electrode is usually in the same range of the molecular film thickness. Therefore, the thinner the molecular layer, the more likely the device becomes shorted. Consequently, to obtain a high yield of working devices, much attention should be paid to carefully control the quality and roughness of every film.

Supplementary Table 1 | Comparison of different techniques for creating sub-10 nm thick molecular devices.

Techniques	Key principle	Efficiency[a]	Structure stability	Addressability[b]	Fabrication scalability	Fabrication difficulty	Ref.
Conductive AFM	Metal-coated AFM tip	Low	Low	Difficult	No	Low	12
Crossed wire junction	Lorentz force	Low	Low	Easy	No	Low	13
Liquid metal contact	Surface tension	Low to medium	Low	Difficult	Possible (PDMS channel)	Medium	14, 15
Suspended nanowire junctions	Dielectrophoretic trapping	Medium	High	Difficult	Possible (randomly distributed)	Medium	16, 17
E-beam deposited carbon film	E-beam deposition	Low to medium	High	Easy	High	Low	18, 19
Surface-diffusion-mediated deposition	Atom diffusion	High	High	Difficult	High	High	10
Nanoskiving	Edge lithography	High	High	Easy	High	Medium to high	20
Pore + metal evaporation	Photolithography, metal evaporation	Low	High	Easy	High	High	21
Pore + conductive carbon/polymer	Photolithography, spin coating	High	High	Easy	High	High	11, 22
Lift-and-float approach	Liftoff and floating	Medium	High	Difficult	Possible (polymer-assisted)	Low	23

(Continued Supplementary Table 1)

Techniques	Key principle	Efficiency[a]	Structure stability	Addressability[b]	Fabrication scalability	Fabrication difficulty	Ref.
Metal transfer printing	PDMS Stamp	Medium to high	High	Easy	High (relying on the stamp)	Medium	24
Graphene transfer	Adhesive tape	Medium	High	Easy	Possible	Medium	25
Rolled-up soft contact	Photolithography, stress release	Medium to high	High	Easy	High (full integration)	High	This work

a, Efficiency: Sum of massive fabrication capability and yield.

b, Addressability: Sum of the capability to settle the molecules, to locate the top electrodes, and to measure the molecular junctions.

Supplementary Note 2. Fabrication process

The molecular devices were integrated on glass wafers. The rolled-up soft-contact was realized by making the strained Au/Ti/Cr nanomembrane roll up and eventually bond back to the molecular layer/bottom electrode. First, a finger-like mesa structure that would support the bottom electrode was formed by etching ~400 nm of the SiO_2 layer in buffered HF solution (Supplementary Figure 1a). After that, a sacrificial layer of GeO_x (oxidized from 20 nm Ge), was patterned (Supplementary Figure 1b), followed by the deposition of metallic nanomembrane Au/Ti/Cr (5/15/20 nm), as shown in Supplementary Figure 1c. The Ti/Cr interface creates a strain gradient and the Au layer acts as the top electrode to the organic layer after rolling. Then, the substrate surface was conformally covered by a passivation layer AlO_x (10 nm) via atomic layer deposition (ALD), as shown in Supplementary Figure 1d. Afterwards, a PEDOT:PSS:AgNWs layer via spin-coating and a Ag film (200 nm) via E-beam deposition were sequentially deposited (Supplementary Figure 1e). The former acts as the bottom electrode after patterning, while the latter is adopted to assist the pattern of the conductive polymer by wet etching and O_2 plasma (Supplementary Figure 1f). After removing the undesirable PEDOT:PSS:AgNWs region, the passivation layer AlO_x was exposed on which windows were opened by 3.3% HF solution for contact pads deposition and rolling direction control (Supplementary Figure 1g). Next, in sequence, a pair of contact pads consisting of Cr/Au (10/50 nm) was patterned to enable electrical measurement, overlapping the area shown in Supplementary Figure 1h. Prior to the deposition of the molecules, the PEDOT:PSS:AgNWs layer on the fingertip was released by etching away the top Ag film (Supplementary Figure 1i). The pattern used to define the etching area can also be applied for molecular layer deposition, and in this work, the deposition was carried out using low temperature evaporation (LTE) in vacuum with a base pressure of 10^{-7} mbar. During deposition, the rates of CuPc and C_{60} were kept at approximately 0.004 nm/s and 0.009 nm/s, respectively, and the substrate was maintained at room temperature (Supplementary Figure 1j). After executing the lift-off process, the wafer was transferred into pure, deionized H_2O for rolling. In this case, the strained metallic nanomembranes curled up because the GeO_x sacrificial layer was slowly removed along the trench in water. The rolling process stopped at the end line of the $GeOx$ layer (Supplementary

Figure 1k and 1l). As a result, the rolled-up nanomembranes contacted the molecular ultrathin film from the top, acting as a top electrode without damaging the molecules.

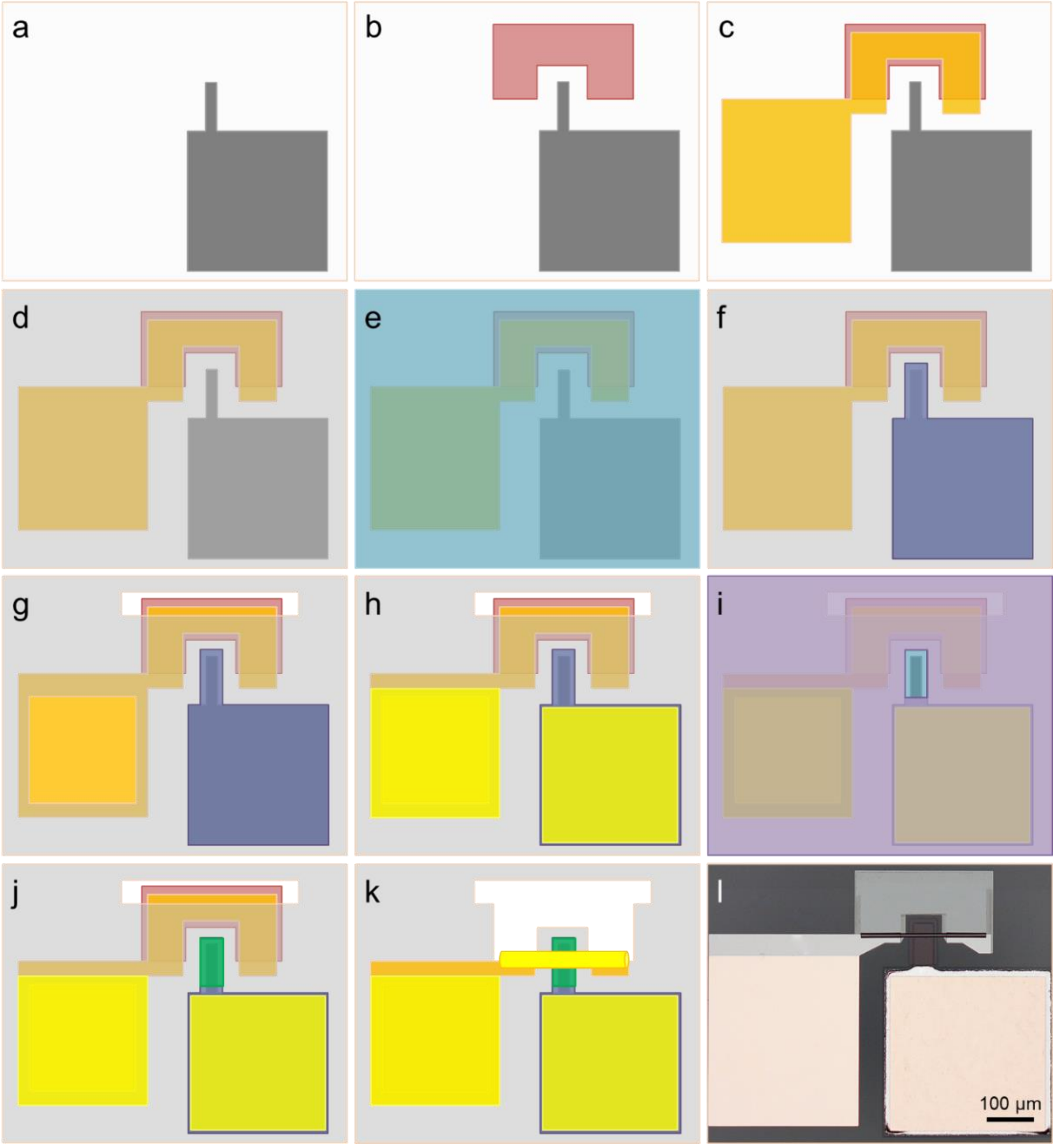

Supplementary Figure 1 | Fabrication process of the molecular devices based on rolled-up soft-contact. a, Patterning finger mesa by HF etching. **b**, Sacrificial layer Ge. **c**, Strain layers Au/Ti/Cr. **d**, Passivation layer Al_2O_3 by ALD deposition. with opened windows. **e**, Successive deposition of PEDOT:PSS:AgNWs layer via spin coating and Ag film (200 nm) via E-beam vapor deposition. **f**, Patterning PEDOT:PSS:AgNWs film by wet etching. **g**, Opening windows in Al_2O_3 layer by HF etching. **h**, Contact pads of Cr/Au. **i**, Opening windows in the remaining PEDOT:PSS:AgNWs film for molecules. **j**, Molecular layer depletion. **k**, Rolling in water. **l**, Optical image of the final rolled-up device.

Supplementary Note 3. Thickness confirmation

During the growth of the molecules, the film thickness was monitored with a quartz crystal microbalance. To determine the actual thickness of each layer, a negative tone of photoresist AZ 5214E was applied. After development, the exposed areas remain with sloped walls (as shown in Supplementary Figure 2a, in purple), which could act as a mask to define the region for the deposited layer. The two LTE sources for ejecting the evaporated molecules are located on the left (for CuPc) and right (for C_{60}) sides, respectively, i.e., not exactly below the substrate. Therefore, there is a shift between the designed region and the deposited area due to the oblique angle deposition through the photoresist mask, as shown in Supplementary Figure 2a. As a result, a small step (i.e., non-overlapping region) can be obtained at each margin of the CuPc/C_{60} hybrid layer, which can be used to determine the real thickness, as shown in Supplementary Figure 2b-e. By this method, the nominal CuPc (5 nm)/C_{60} (5 nm) hybrid layer is measured to be 4.7 nm and 5.1 nm, respectively. While for the case of CuPc (20 nm)/C_{60} (20 nm) hybrid layer, the real thicknesses of CuPc and C_{60} are approximately 18.5 nm and 20 nm, respectively. Since the deviation is small, we still use the nominal height to label the molecular devices.

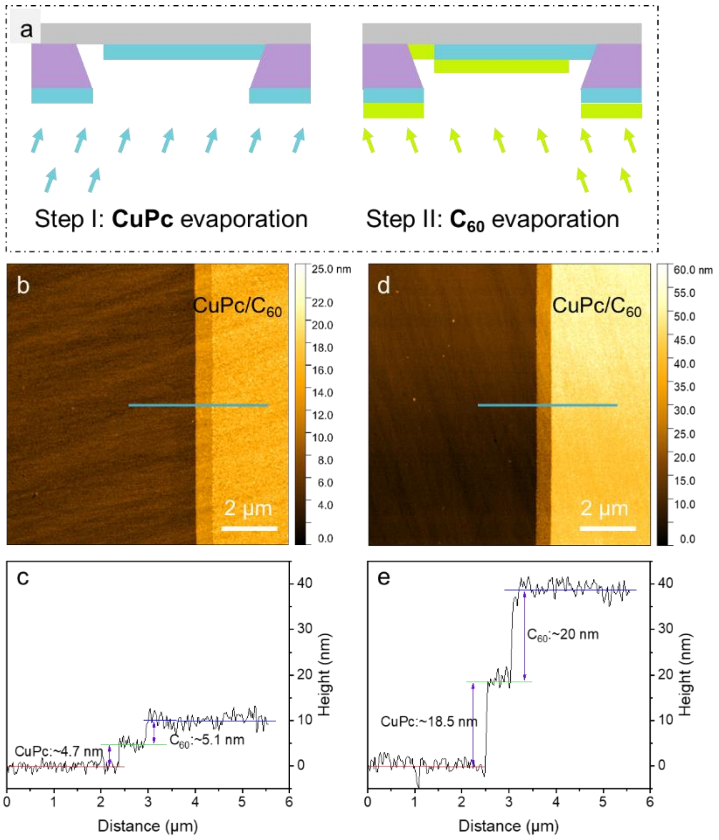

Supplementary Figure 2 | Confirmation of the actual thickness of each molecular layer. a, Schematic illustration of the oblique angle deposition of molecules through the photoresist mask. The grey, purple, blue, and green regions represent the substrate, photoresist pattern, CuPc, and C_{60}, respectively. The arrows indicate the deposition direction. **b** and **c**, AFM image of CuPc (5 nm)/CuPc (5 nm) grown on a wafer and the corresponding height profile of the AFM image. **d** and **e**, AFM image of CuPc (20 nm)/CuPc (20 nm) grown on a wafer and the corresponding height profile of the AFM image.

Supplementary Note 4. Damage-free method to pattern PEDOT:PSS:AgNWs film

PEDOT:PSS is a well-developed ionic-electronic mixed conductive polymer, which is comprised of a negatively charged PSS-rich matrix and positively charged PEDOT-rich cores[26, 27]. Based on this charged property, PEDOT:PSS can act as an ion reservoir to supply mobile ions. Due to the direct contact with photoresist could seriously degrade the conductivity of the PEDOT:PSS:AgNW layer and cause contamination, an Ag protecting film was deposited on the conductive polymer to assist the pattern transfer, as shown in Supplementary Figure 3. Briefly, 200 nm of Ag film was deposited on top of the spin-coated PEDOT:PSS:AgNWs layer via E-beam deposition. Then, a conventional photolithography process was performed with AZ 5214E (Microchemicals GmbH) as the photoresist. As described in Supplementary Note 2, there are two steps (i.e., Supplementary Figure 1f and 1i) that involve the Ag film assisted etching process. For patterning the bottom electrode, both the unwanted Ag film and the underneath PEDOT:PSS:AgNWs layer should be removed. While for opening window for molecule deposition, only the Ag film at the fingertip will be etched. The removal of the Ag film was realized by wet etching with a mixed acidic solution, H_3PO_4:HNO_3:CH_3COOH:H_2O with a ratio of 3:3:23:1. After that, the PEDOT:PSS:AgNWs layer was exposed, which can be easily removed by O_2 plasma. To avoid contamination from the photoresist after etching, the used AZ 5214E pattern was remained for the subsequent molecular layer deposition (because if fresh photoresist is spin-coated, the exposed PEDOT:PSS:AgNWs layer will be damaged).

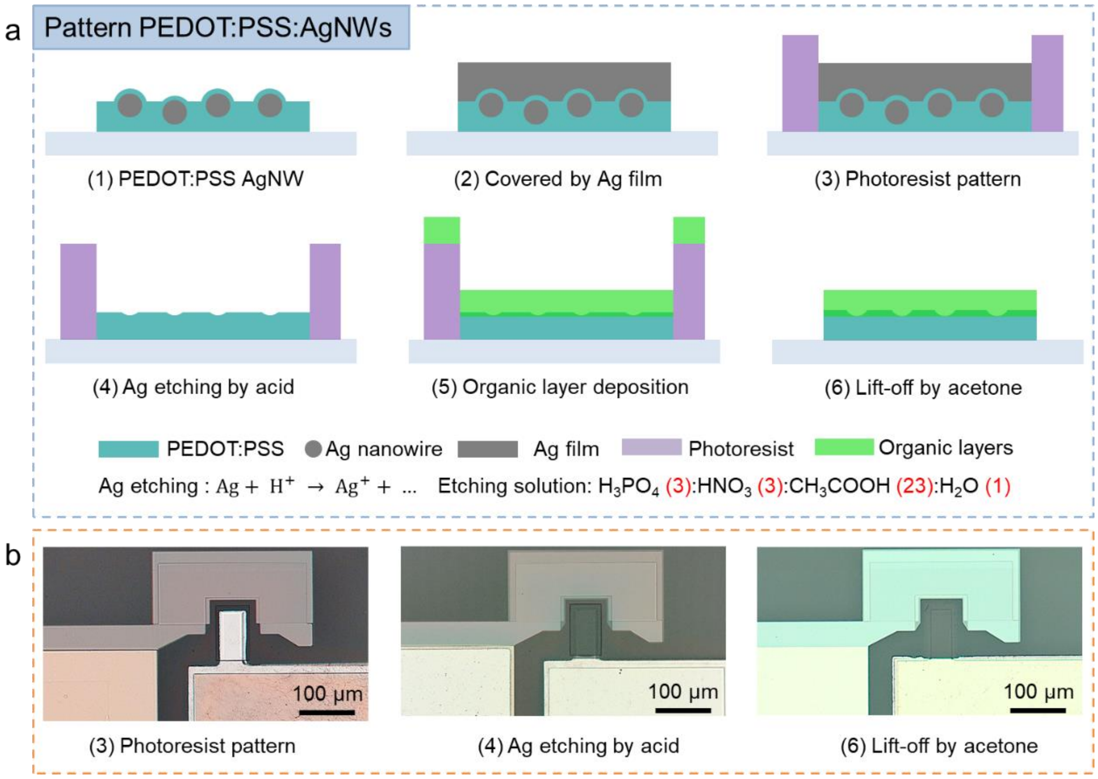

Supplementary Figure 3 | Schematic presentation of patterning the PEDOT:PSS:AgNWs film. a. The main patterning process, in which a Ag film was used to transfer the designed structure to a PEDOT:PSS:AgNWs film with a conventional photolithography technique. **b.** The corresponding optical images of the critical steps mentioned in **a**.

Supplementary Note 5. Effects of etching PEDOT:PSS:AgNWs film

After etching, the PEDOT:PSS:AgNWs film is densified (Supplementary Figure 4), which is consistent with the thickness shrinkage of a PEDOT:PSS film from 50 nm to 30 nm (Supplementary Figure 5). The AFM topography images were obtained by a Bruker's atomic force microscope (Dimension Icon®) with tapping mode, and the thickness was measured by a Bruker's Dektak XTL™ stylus profilometer. On the other hand, the conductivity decreases from 3000 S/cm to 700 S/cm (Supplementary Figure 5), but still can work as a qualified electrode. The conductivity is confirmed by the typical "four probe method". Moreover, the etched PEDOT:PSS:AgNWs film also shows good transparency (Supplementary Figure 6). The transparency is measured with a light source and a photodetector. Therefore, the etched PEDOT:PSS:AgNWs film can be an excellent transparent electrode material.

Another interesting phenomenon is that based on the relative atomic content derived from the XPS spectrum (Supplementary Table 2), longer etching leads to a higher Ag 3*d* content which can be attributed to longer ion adsorption (the ion source mainly stems from the dissolution of the 200 nm Ag protective layer), indicating that Ag is most likely in the form of ions. Because if the Ag element exists in the form of metallic Ag residual, the Ag 3*d* signal should become weaker after longer etching, which is contrary to the experimental results. In fact, the etching rate of the mixed acid solution is very fast, as the 200 nm Ag film could be totally etched within 10 s.

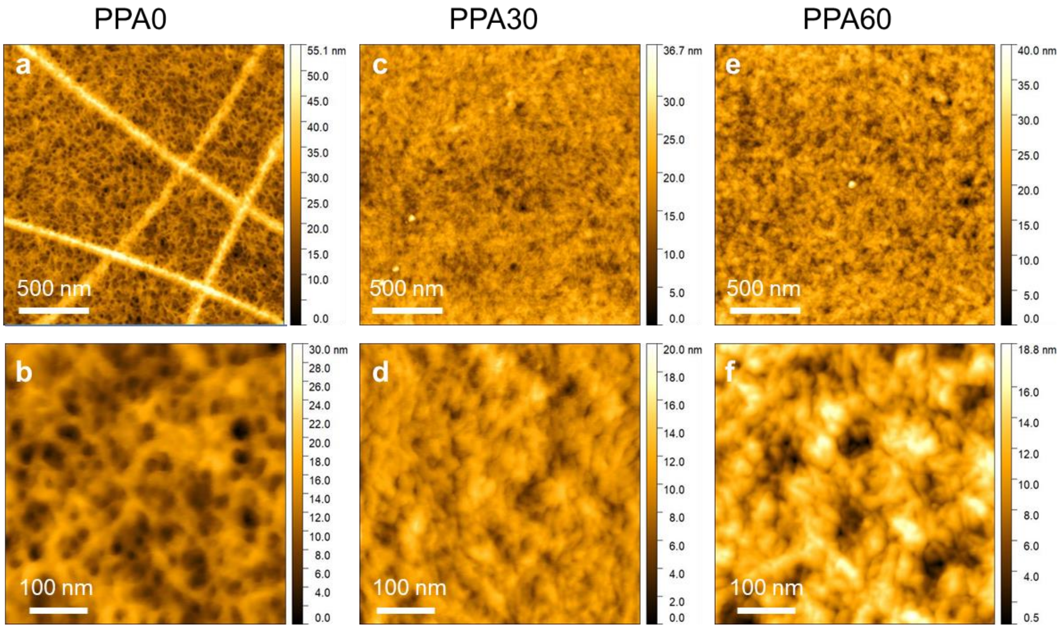

Supplementary Figure 4 | AFM images of the etched PEDOT:PSS:AgNWs films. a and **b**, The pristine film. **c** and **d**, The film etched with 30 s. **e** and **f**, The film etched with 60 s.

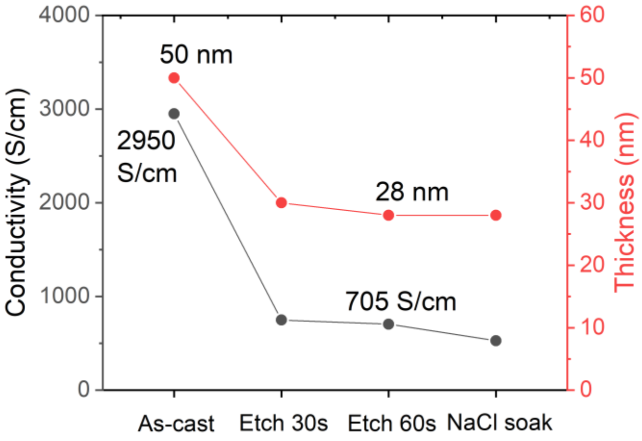

Supplementary Figure 5 | Conductivity and thickness of the etched PEDOT:PSS:AgNWs films

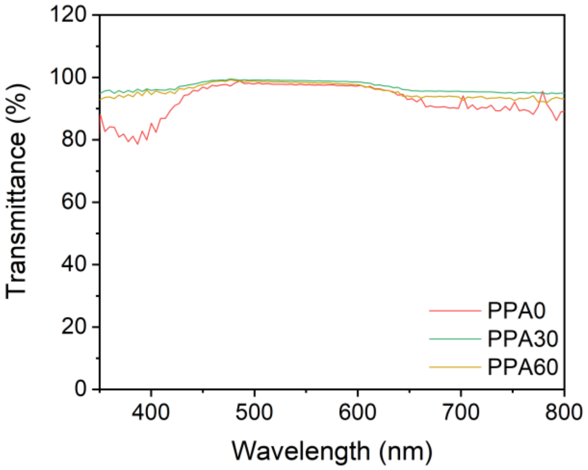

Supplementary Figure 6 | Transmittance spectra of the etched PEDOT:PSS:AgNWs films.

Supplementary Table 2 | The work function (WF) and the relative atomic content derived from the photoelectron spectroscopy measurement under different wet etching conditions.

Sample	WF (eV)	C 1*s* (%)	N 1*s* (%)	S 2*p* (%)	Ag 3*d* (%)
PPA30	4.8	88.73	1.75	9.28	0.24
PPA60	4.5	88.56	1.82	9.26	0.37

Supplementary Note 6. Traditional photodiodes and photomultiplication photodiodes

Photodiodes, a branch of photodetectors that convert incident photons into electrical signals, have plenty of important applications similar to other photodetectors, such as image sensing, optical communication, safety/environmental monitoring, and chemical/biological detection. One important index is the external quantum efficiency (EQE), which is initially defined as the ratio of the converted electron to the incident photon[28]

$$\mathrm{EQE} = \frac{\text{converted electron}}{\text{incident photon}} = \frac{(J_L - J_D)hv}{eP_{in}} \times 100\%$$

where J_L, J_D, e, P_{in}, and hv are the photocurrent density, dark current density, elementary electron charge, light power density, and incident photon energy, respectively. Based on the EQE value, photodiodes can be divided into two categories: traditional photodiodes[29] and photomultiplication photodiodes[30], as depicted in Supplementary Figure 7a. Specifically, taking a p-n junction-based traditional photodiode as the prototype (as shown in the left panel), the photophysical process mainly involves three steps[31]: (1) exciton generation by photon adsorption in the donor and/or acceptor materials due to the photoelectric effect; (2) separation of the excitons into free-charge carriers (holes and electrons) at the donor/acceptor interfaces; and (3) charge carriers transport and extraction to the electrodes. The photocurrent is attributed to the increase in electrical conductivity resulting from the increased carrier density in the photoactive channel when photons are absorbed. Theoretically, an adsorbed photon can excite at best one electron-hole pair, hence, the calculated EQE cannot exceed 100%. In this kind of photodiodes, the both electrode contacts are normally ohmic-like, so that the photogenerated holes and electrodes are readily collected by the anode and cathode, respectively.

When one of the electrode contacts of the p-n junction-based photodiode is Schottky rather than ohmic, i.e., a large energy offset exists between the electrode Fermi level (E_F) and the HOMO or LUMO of the photoactive material, the photogenerated charge carrier collection efficiency could be seriously decreased, leading to a small photocurrent as well as a low EQE. In fact, it is still possible to obtain high EQE photodiodes with Schottky contact. In addition to the Schottky photodiode (with a similar working principle with p-n junction photodiode), another

interesting type is the well-known photomultiplication photodiode which depends on improved charge injection induced by the interfacial traps[32], as shown in Supplementary Figure 7b. Trap states can be introduced as the second phase (e.g., quantum dots, nanoparticles, dyes) dispersed in the photoactive matrix layer or as the interfacial layer between the electrode and photoactive layer. Normally, there is a large difference in HOMO or LUMO between photoactive and trapping materials, therefore, photogenerated holes or electrons can be captured[33]. One type of photogenerated charge carriers trapped in the trap states near the electrode could produce a Coulomb field, which induces interfacial band bending to assist the tunneling injection of the opposite charge carriers from the external circuit[34, 35]. Trap-assisted injection under light illumination is not strictly limited to the photogenerated carrier density but relies on the number of trapped carriers near the electrode, so a photomultiplication phenomenon with an EQE higher than 100% could happen. Photomultiplication is desirable for highly sensitive photodetectors with excellent detection capability, which can simplify the photodetection system with no need for a preamplifier circuit. It should be mentioned here that photomultiplication photodiodes just borrow the existing EQE to describe the sensitivity, not the same as traditional photoelectric effect photodiodes. In fact, the EQE in photomultiplication photodiodes can be regarded as the ratio of the secondary electron from the trap-assisted injection to the incident photon. Due to the intrinsic feature of electric-field-driven trapping/detrapping, photomultiplication photodiodes are suffering two problems: high driven voltage and slow photo-response.

Usually, photodiodes based on the pure photoelectric effect should apply thick active layers to avoid inefficient photon absorption. However, we successfully realized molecular photodiodes with high photosensitivity based on several nanometer-thick molecule ensembles, which is ascribed to the trap-assisted injection, instead of a carrier density increase in the active layer.

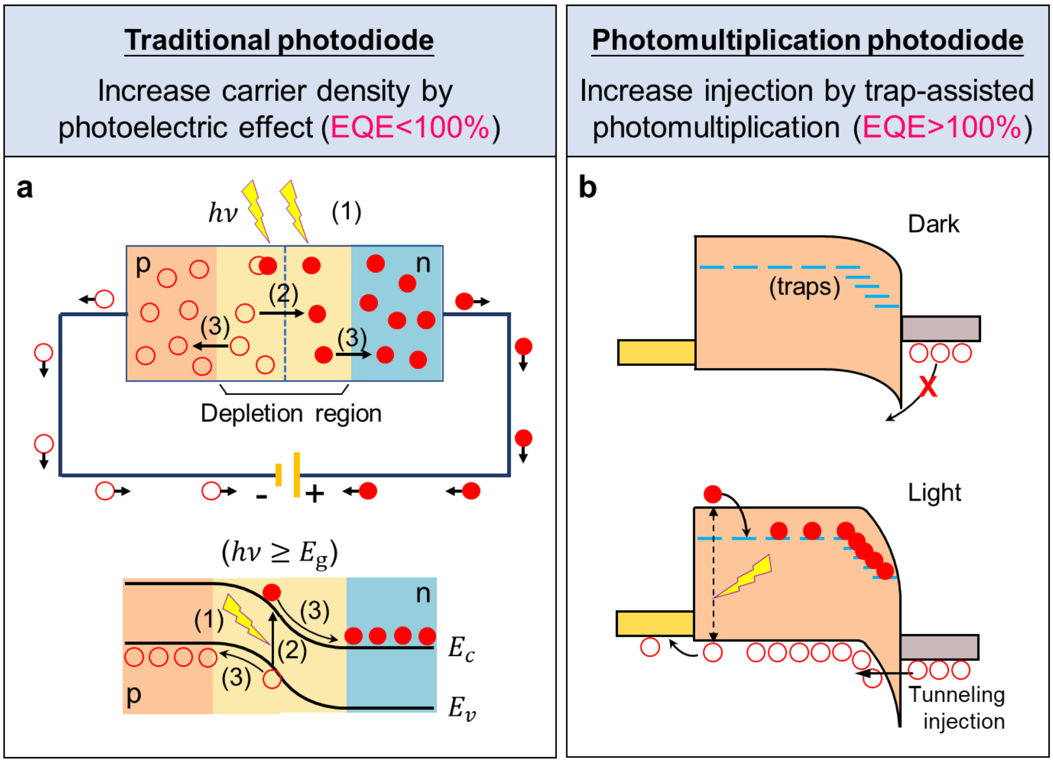

Supplementary Figure 7 | Schematic diagrams of two kinds of photodiodes categorized according to the external quantum efficiency (EQE). a, Traditional photodiodes that rely on the increased carrier density by photogenerated carriers have EQE less than 100%, which mainly involve the conventional three steps: (1) photon adsorption, (2) exciton separation, and (3) holes/electrons collection. **b,** Photomultiplication photodiodes have EQE exceeding 100% due to the trap-assisted photomultiplication effect. The red round and red circular ring represent electron and hole, respectively.

Supplementary Note 7. Photomultiplication performances

The photoresponses to single-wavelength light are also characterized by three individual laser light sources (5 mW/cm^2), i.e., 459 nm, 532 nm, and 630 nm, respectively. Calculated from the *I-V* characteristics of the molecular device, PPA30/CuPc (3 nm)/C_{60} (3 nm)/Au tube, in the dark and under illumination (Supplementary Figure 8), the EQE values at -1 V with wavelengths of 459 nm, 532 nm, and 630 nm are 21124%, 29051%, and 79241%, respectively. For PPA30/CuPc (3 nm)/C_{60} (3 nm)/Au tube under 630 nm laser illumination, the calculated EQE can exceed 100% at only -0.1 V (Supplementary Figure 9a), which means that the applied voltage for photomultiplication is greatly decreased due to the ultrathin organic heterojunction.

The time-dependent photoresponses are measured, as shown in Supplementary Figure 9b with a 630 nm laser light source (5 mW/cm^2). Both the response and recovery processes take on a time scale of seconds (Supplementary Figure 9), much slower than traditional photodiodes (in a range of nanoseconds to milliseconds), which come from the intrinsic feature of electric-field-driven trapping/detrapping. The photocurrent is mainly generated by trapped holes in the C_{60} layer near the Au tube electrode (cathode), which assists electron injection. The accumulation of an adequate number of trapped holes to trigger extensive band bending at the active layer/cathode interface and facilitate electron injection requires a relatively long time, resulting in a relatively long rise time. The slow decay process for the photocurrent is attributed to the slow recombination process for the trapped holes in C_{60} near the cathode.

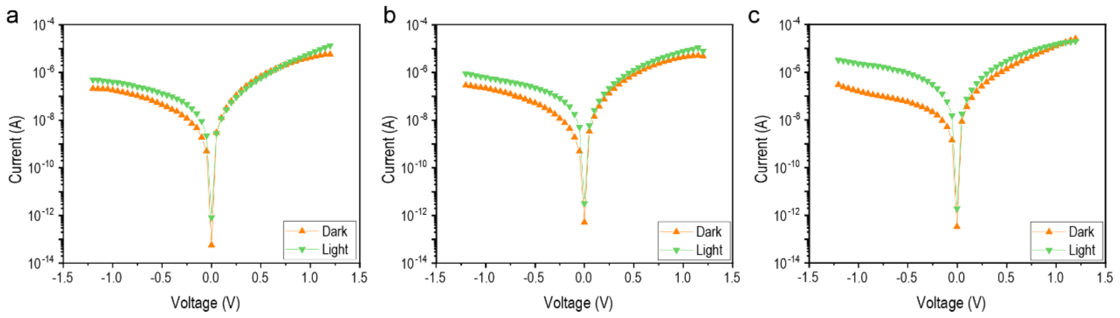

Supplementary Figure 8 | Photoresponses of PPA30/CuPc (3 nm)/C_{60} (3 nm)/Au tube to single-wavelength light. a, Under illumination of 459 nm laser light sources (5 mW/cm^2). **b**, Under illumination of 532 nm laser light sources (5 mW/cm^2). **c**, Under illumination of 630 nm laser light sources (5 mW/cm^2).

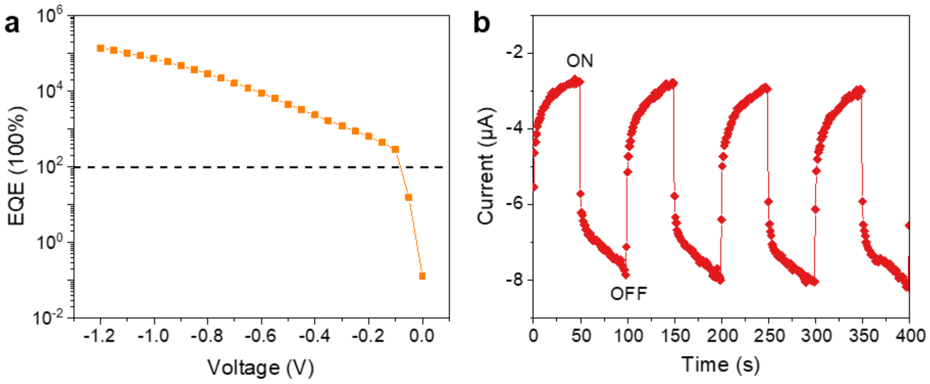

Supplementary Figure 9 | Photoresponse of PPA30/CuPc (3 nm)/C_{60} (3 nm)/Au tube by a 630 nm laser light source (5 mW/cm^2). a, Voltage-dependent EQE. At -0.1 V, the calculated EQE already exceeds 100%. **b**, Time-dependent photoresponse at -0.5 V.

Supplementary Note 8. Transition voltage spectroscopy

Transition voltage spectroscopy (TVS) is a useful tool to gain insights into the energy alignment in molecular junctions by providing an indirect measure of the position between the electrode Fermi level (E_F) and the frontier orbital level (HOMO or LUMO) of molecules[36]. It is of great significance to investigate the charge transport mechanisms and realize the functions of molecular junctions. With TVS analysis, an inflection point (transition voltage V_T) can be extracted from a plot of In (I/V^2) vs. $1/V$, which is proportional to the effective charge injection barrier height (Φ) and is consistent with a transition from direct tunnelling (DT) to field emission (also known as Fowler-Nordheim tunnelling (FNT))[37].

At a low-bias region, direct tunnelling dominates where the relationship between current (I) and voltage (V) can be described as follows[38]:

$$I \propto Vexp\left[-\frac{2d\sqrt{2m_e\Phi}}{\hbar}\right]$$

where d is the thickness of the molecular layer; m_e is the electron effective mass; and $\hbar$ is the Planck constant. Therefore, $1/V$ is logarithmically scaled with In (I/V^2), i.e., In (I/V^2) $\propto$ In ($1/V$). At a high-bias region, for the applied bias exceeding the transition voltage V_T, I-V follows the Fowler-Nordheim tunnelling relation[38]:

$$I \propto V^2exp\left[-\frac{4d\sqrt{2m_e\Phi^3}}{3\hbar qV}\right]$$

where q is the electronic charge. From the above equation, it can be deduced that In (I/V^2) against $1/V$ has a linear relationship with a negative slope.

Returning to our work, in order to investigate the thickness-dependent charge transport mechanism of photomultiplication photodiodes under dark and illumination conditions, TVS is applied to fit the data at reverse bias (i.e., the bottom electrode is positively biased), as shown in Supplementary Figure 10. We observe controllable thickness and light dependences of V_T in the molecular junction, which can be well explained by trap-assisted injection. Specifically, without illumination, only the device based on PPA30/CuPc (3 nm)/C_{60} (3 nm)/Au tube exhibits

two distinct regions with the transition voltage point located at 0.8 V. This phenomenon indicates that, on the one hand, field emission occurs within PPA30/CuPc (3 nm)/C_{60} (3 nm)/Au tube, and on the other hand, CuPc (5 nm)/C_{60} (5 nm) and CuPc (20 nm)/C_{60} (20 nm) are too thick to trigger Fowler-Nordheim tunnelling due to the relatively low electric field at the C_{60}/Au interface with an applied voltage ranging from 0 to -1.2 V. Under illumination, the photogenerated excitons will be dissociated into charge carriers (electrons and holes) at the CuPc/C_{60} interface, and the photogenerated electrons will be transported through CuPc and then collected by the PPA30 electrode at reverse bias. While the photogenerated holes will be trapped in C_{60} due to 1 eV of the highest occupied molecular orbital (HOMO) difference between CuPc and C_{60}. According to the working principle of photomultiplication photodiodes, only sufficient holes trapped in C_{60} near the Au electrode can generate a strong Coulomb field to cause interfacial band bending for efficient electron tunneling injection from the external circuit, thus resulting in a high photocurrent. Therefore, the hole-trapping layer C_{60} should be thin enough; otherwise, the induced local electric field cannot reach the Au electrode and cannot obviously improve the electron injection from the Au electrode to C_{60}, which is consistent with the fitting results shown in Supplementary Figure 10. For the case based on CuPc (3 nm)/C_{60} (3 nm), the transition voltage V_T decreases from 0.8 V to 0.5 V after switching on the light (Supplementary Figure 10a). Moreover, an inflection point at 0.9 V of the device based on CuPc (5 nm)/C_{60} (5 nm) is observed under illumination (Supplementary Figure 10b). These two phenomena imply that the trapped holes within 3 nm/5 nm C_{60} can effectively make the Schottky barrier (between C_{60} and Au) thinner, resulting in the decrease in transition voltage and the consequent photomultiplication effect. On the contrary, the trap-induced band bending in 20 nm C_{60} is negligible so that there is still no transition under illumination (Supplementary Figure 10c) as well as no improved electron injection.

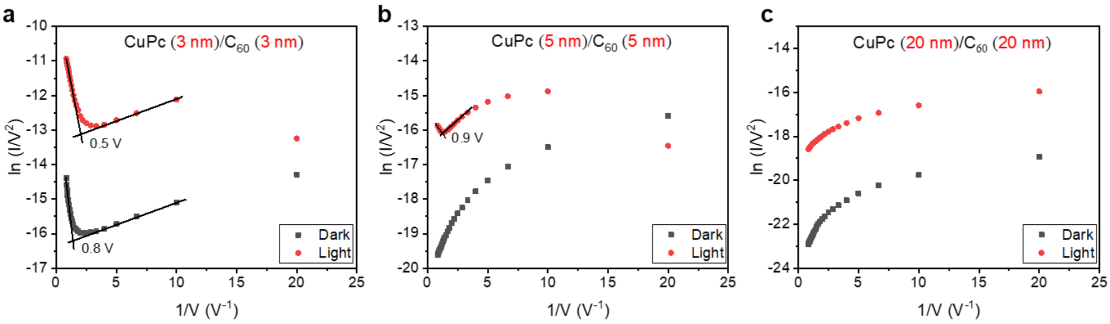

Supplementary Figure 10 | Fowler-Nordheim fitting of the *I*-*V* data at reverse bias. a, The 1/V ~ ln (I/V^2) plot of the device based on CuPc (3 nm)/C_{60} (3 nm). **b**, The 1/V ~ ln (I/V^2) plot of the device based on CuPc (5 nm)/C_{60} (5 nm). **c**, The 1/V ~ ln (I/V^2) plot of the device based on CuPc (20 nm)/C_{60} (20 nm). The thickness-dependent fitting proves that the trap-assisted injection only works when the molecular ensemble is thin enough.

Supplementary Note 9. Statistics

Statistics of the molecular devices are shown in Supplementary Figure 11. As extracted from Supplementary Figure 11a-c, the yields of the molecular devices based on CuPc(3 nm)/C_{60}(3 nm), CuPc(5 nm)/C_{60}(5 nm), and CuPc(20 nm)/C_{60}(20 nm) are 39.7% (=27/68), 48.5% (=33/68), and 63.2% (=43/68), respectively. This trend in yield versus thickness originates from the fact that the thinner the molecular layer, the more likely the device becomes shorted, because the roughness (~ 2nm) of the top tubular and bottom finger electrodes is in the same range of the molecular film thickness, which can shrink the effective gap between the two electrodes. Statistics of the device performance are also provided in terms of the rectification ratio, ON/OFF ratio, ON-state current, and switching voltage, as shown in Supplementary Figure 11d-g. Since the hysteretic behaviour of PPA60/CuPc(20 nm)/C_{60}(20 nm)/Au is not remarkable, only the parameters of molecular devices based on CuPc(3 nm)/C_{60}(3 nm) and CuPc(5 nm)/C_{60}(5 nm) are presented.

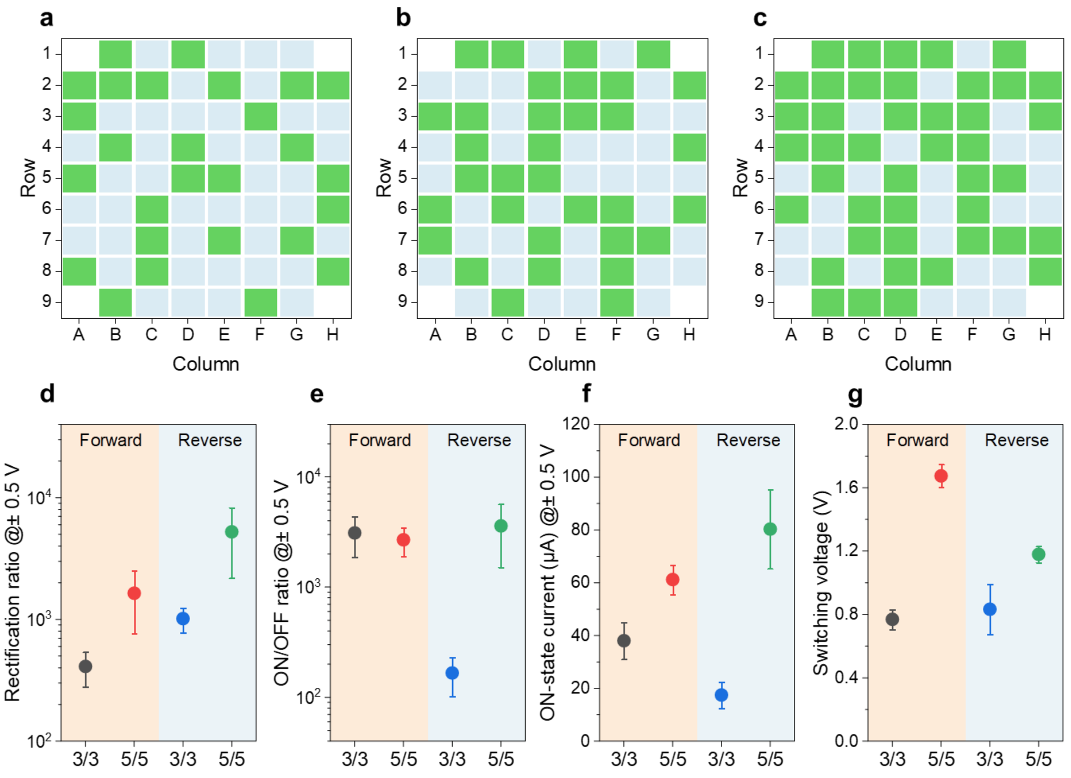

Supplementary Figure 11 | Statistics. a-c, Distribution maps of the molecular devices based on CuPc(3 nm)/C_{60}(3 nm), CuPc(5 nm)/C_{60}(5 nm), and CuPc(20 nm)/C_{60}(20 nm), respectively.

The green and gray squares represent successful and failed devices on the sample substrates, where a “successful device” refers to a device that exhibits *I-V* hysteresis behaviour and a “failed device” refers to a shorted or open device. **d-g**, Average values of the rectification ratio at ±0.5 V (**d**), ON/OFF ratio at ±0.5 V (**e**), ON-state current at ±0.5 V (**f**), and the switching voltage (**g**). Error bars represent the standard deviation for each parameter of the working devices. “3/3” and “5/5” denote the molecular devices based on CuPc(3 nm)/C_{60}(3 nm) and CuPc(5 nm)/C_{60}(5 nm), respectively. All data are obtained with the bottom finger electrodes grounded. “Forward” and “Reverse” in **d** indicate the forward diode and reverse diode, respectively. “Forward” and “Reverse” in **e-g** indicate the positive and negative voltage regions, respectively.

Supplementary Note 10. Memristive mechanism

SN 10.1 Filamentary vs. barrier modulation

To elucidate the working mechanism, we first compare the *I-V* hysteresis features of our memristors to those of conventional filamentary memristors, as shown in in Supplementary Figure 12. We fabricated a stacked structure of Ni/SiO_2(1 nm)/Al_2O_3(1 nm)/NiFe, which exhibits a typical filamentation-type hysteresis behavior[39, 40] (Supplementary Figure 12a) owing to the formation and rupture of conductive filaments. In this situation the sequence of the *I-V* hysteresis follows HRS→LRS→LRS→HRS, where HRS and LRS are the abbreviations of the high resistance state (i.e., OFF state) and low resistance state (i.e., ON state), respectively. Therefore, the memristor can maintain the ON/OFF state when the bias polarity is switched. However, for the molecular devices in our work (as shown in Supplementary Figure 12d), the *I-V* hysteresis follows the order HRS→LRS→HRS→LRS; i.e., the resistance state changes whenever the bias polarity switches, which is consistent with the behaviour of memristors that rely on a Schottky barrier modulation in perovskite-based devices, such as $BiFeO_3$ (BFO)[41], BaTiO (BTO)[42], $CH_3NH_3PbI_3$ ($MAPbI_3$)[43], and so on. For this kind of perovskite memristors, the observed hysteresis is generally accepted to be caused by ion migration-induced barrier modulation, which is supported by experimental results[42, 43, 44] and numerical simulations[45]. Under an electric field, mobile ions drift in the perovskite layer and accumulate near the electrode surfaces, leading to p-doping in the perovskite near the anode and n-doping near the cathode. Similarly, a reverse bias can flip the doping configuration by forcing positive and negative ions to drift in the opposite direction. The self-doping effect can influence the electrode/perovskite barrier width and therefore increase or decrease the carrier injection capability, resulting in memristive hysteresis. In perovskite materials, the mobile ions originate from lattice defects at the grain boundaries, for example, positively charged I vacancies and negatively charged Pb and MA vacancies in $MAPbI_3$[43].

Additional evidence for clarifying the mechanism is provided by fitting the double logarithmic *I-V* plot with a power law[46], as shown in Supplementary Figure 12. For filamentation-type memristors, the ON state is attributed to the formation of conductive filaments between the two

electrodes, which causes a “short circuit”. Therefore, the charge transport of the On-state is governed by Ohmic conduction, and the corresponding *I-V* plots of the LRS for both negative and positive voltages are characterized by Ohmic behavior ($I \sim V$)[47, 48], i.e., the slope is close to 1 (as shown in Supplementary Figure 12b and 12c). However, for the molecular devices in our work (as shown in Supplementary Figure 12e and 12f) the slopes of the On-state *I-V* plots at negative and positive voltages are calculated to be 2.1 and 3.2, respectively, which deviate significantly from Ohmic behavior. Instead, the linear fitting matches well with perovskite-based memristive switching[42].

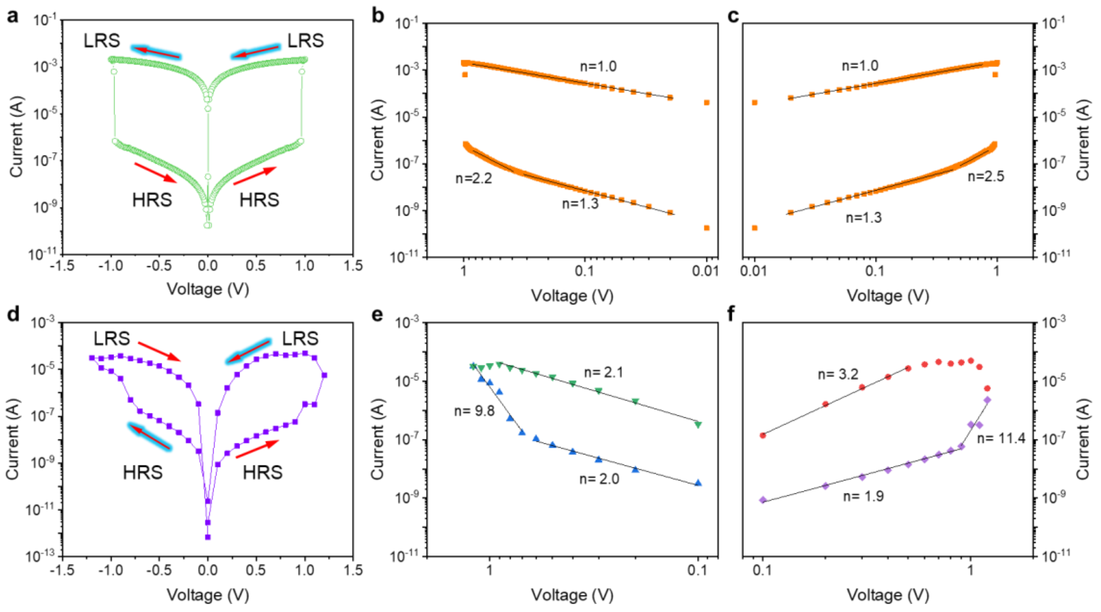

Supplementary Figure 12 | Two types of bipolar memristors according to the *I-V* hysteresis sequence. a, *I-V* hysteresis follows the order of HRS→LRS→LRS→HRS. The example shown here is based on a stacked structure of Ni/SiO_2(1 nm)/Al_2O_3(1 nm)/NiFe. HRS and LRS are the abbreviations of the high resistance state (i.e., OFF state) and low resistance state (i.e., ON state), respectively. **b** and **c**, Double logarithmic fittings of the negative part (**b**) and positive part (**c**) of the *I-V* plot shown in **a**. **d,** *I-V* hysteresis follows the order of HRS→LRS→HRS→LRS. The example shown here is based on our PPA60/CuPc(3 nm)/C_{60}(3 nm)/Au. **e** and **f**, Double logarithmic fittings of the negative part (**e**) and positive part (**f**) of the *I-V* plot shown in **d**.

SN 10.2 Switchable diode effect

The switchable diode effect that usually appears in interface-type perovskite memristors[41, 49] is also observed in our molecular devices (as shown in Supplementary Figure 13), which has not yet been reported in filamentation-type memristors as far as we know. For the virgin state, the current is very small and nearly symmetric over a small bias range (Supplementary Figure 13a); however, when the sweep starts from a higher negative voltage (-1.2 V here), the current increases rapidly in the negative voltage region but increase slowly in the positive voltage region, indicating a reverse diode behavior (Supplementary Figure 13b); whereas, when the sweep starts from a higher positive voltage (+1.2 V here), the current shows a forward diode (Supplementary Figure 13c). Moreover, this switchable diode effect also happened in single-direction sweeping. For example, when sweeping from -1.2 V to 1.2 V alone (Supplementary Figure 13d), the rectification ratio I (-0.5 V)/I (0.5 V) = 800, while sweeping from 1.2 V to -1.2 V generates a ratio I (0.5 V)/I (-0.5 V) = 100 (Supplementary Figure 13e). The mechanism of the switchable diode effect in perovskite devices is still under debate: some researchers claim that it originates from the polarization of mobile ions[43, 44, 50]; while some others claim that it is governed by the polarization of ferroelectric domains[41, 51]. However, both of them agree that the polarized charges (ions or charged centres) under the applied electric field can modify the electrode/perovskite Schottky barriers, leading to switchable diode behavior. It should be pointed out that in our case both CuPc and C_{60} are not ferroelectric materials.

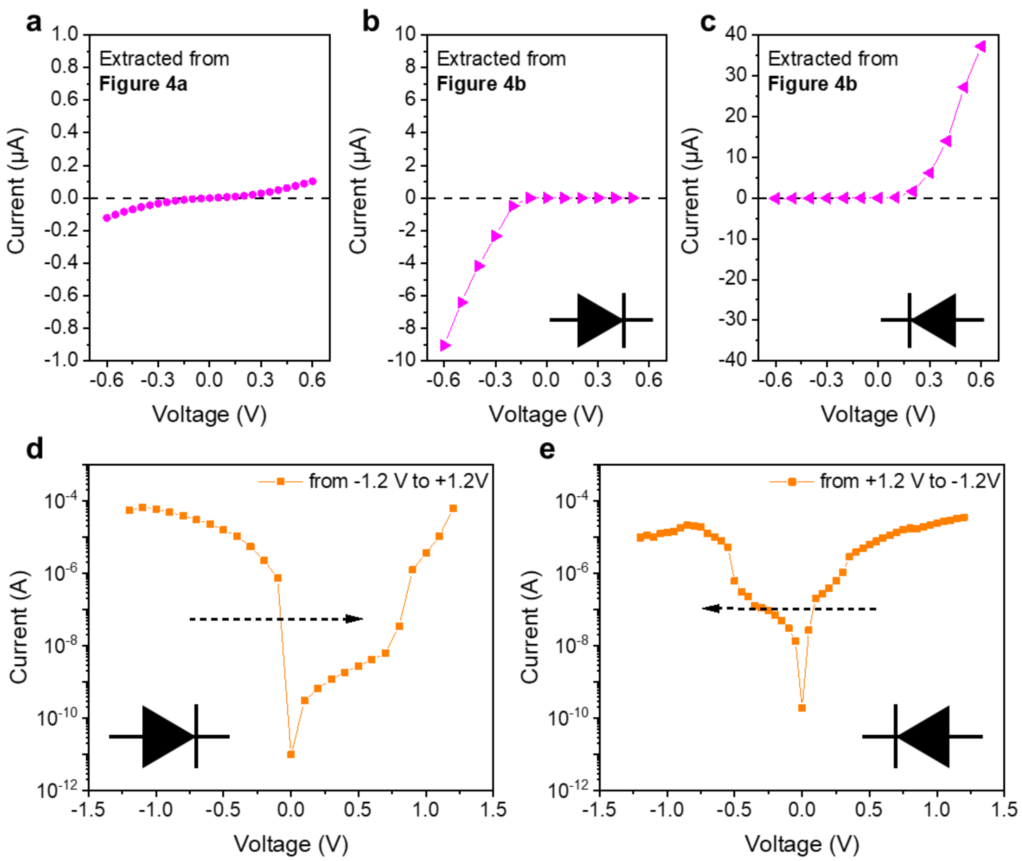

Supplementary Figure 13 | Voltage-polarity-dependent rectifying behavior. a, Virgin state extracted from Figure 4a in the manuscript. **b** and **c**, Reverse diode (**b**) and forward diode (**c**) behaviors extracted from Figure 4b in the manuscript. The black symbols represent diodes. **d**, Single sweep from PPA60 bottom electrode to Au tubular top electrode, resulting in a diode-like rectifying behavior with negative voltage as the forward bias. **e**, Single sweep from Au tubular top electrode to PPA30 bottom electrode, resulting in a diode-like rectifying behavior with positive voltage as the forward bias. The bottomPPA60 electrode is grounded.

SN 10.3 Photoresponse of the molecular memristors with *I-V* hysteresis

As shown in Supplementary Figure 14, compared to the dark condition, the *I-V* hysteresis almost disappears under illumination. The shrinkage of the *I-V* hysteresis under illumination has also been reported in perovskite-based solar cells and photodetectors due to ion-involved phenomena[51, 52]. Most of the mobile ions drift toward the contacts under a high external electric field, and in turn, the ion accumulation impacts the electron and hole distributions (i.e., ion accumulation-induced doping effect), which is deleterious to the performance of the devices in

the photovoltaic and light detection fields. Owing to the presence of a very large electron/hole concentration at the interface (where photogenerated holes/electrons would be extracted), these accumulated ions at the electrode interfaces can act as electron-hole recombination centers (i.e., traps) to enhance the interfacial charge recombination during the collection of photogenerated carriers[53]. As a consequence, the barrier modulation effect induced by ion accumulation is weakened, hence, the photocurrent is reduced[54] and the *I-V* hysteresis reduces under illumination after polarization. Furthermore, the presence of ions at the interfaces can lead to chemical reactions at the contacts, which could also modify the extraction properties. Back to our case, it is supposed that both the enhanced surface recombination and chemical reaction contribute to the shrinkage of the *I-V* hysteresis. Because after turning off the light, the *I-V* hysteresis in the dark is severely reduced, especially for the negative part, which is possibly ascribed to the lack of Ag ions owing to the photoreduction of Ag ions with the help of photogenerated electrons.

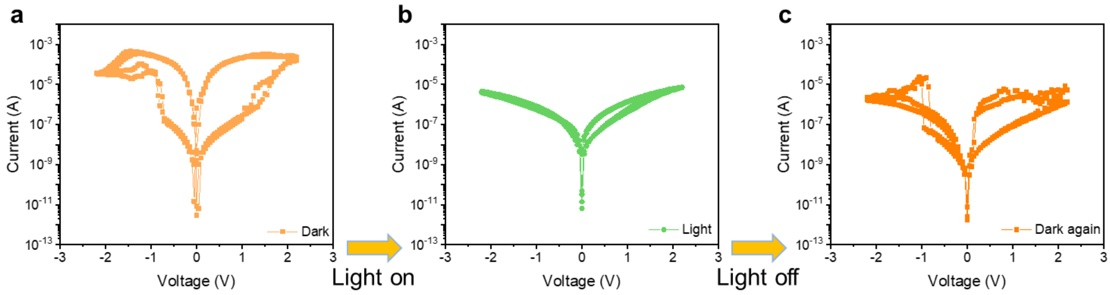

Supplementary Figure 14 | Photoresponse of the molecular device based on PPA60/CuPc (5 nm)/C_{60} (5 nm)/Au tube. a, *I-V* characteristics in dark. **b**, *I-V* characteristics under illumination. **c**, *I-V* characteristics in the dark again.

Supplementary Note 11. Ion doping-assisted injection

In addition to trap-induced band bending under illumination, the junction barrier at the electrode/organic semiconductor interface can also be effectively modulated by ion polarization under an electric field[55, 56, 57], as depicted in Supplementary Figure 15. After applying an external bias, the first step involves a redistribution of the ions in the active layer. Driven by the electric field, anions and cations migrate to the respective electrode interfaces and

accumulate to form thin sheets (ranging from 1 to 6 nm[55, 58]) of uncompensated charge referred to as electric double layers (EDLs). Then, holes and electrons are injected to compensate the accumulated ions of opposite sign (i.e., anions and cations, respectively) present at the electrode interfaces, which are identical to electrochemical doping. Consequently, anion- and cation-accumulated regions are p- and n-doped, respectively[59]. If the ion supply is not limited and/or the active channel is thin enough, the p- and n-doped zones can continue to grow with operating time until they eventually make contact, i.e., evolve from p-i-n junction to p-n junction[43]. At the same time, the compensating holes (electrons) give rise to strong downward (upward) band bending in the semiconductor near the cathode (anode) due to the doping effect, resulting in a narrowed barrier width and enhanced hole (electron) tunneling injection[55].

The electrochemical doping process is the working principle of light-emitting electrochemical cells[60] and ferroelectric memristors[43]. Normally, the active materials contain mobile ions that originate either from the electrolyte within the polymer matrixes or from the undercoordinated ionic defects (such as oxygen vacancies) of perovskites[44], which is the prerequisite for ion polarization. Briefly, for light-emitting electrochemical cells (LECs), the injected holes and electrons migrate through the electrochemically doped regions and recombine within, or in close proximity to, the undoped junction, causing the emission of light. As one of the simplest kinds of electroluminescent devices, LECs usually operate with low voltages and in turn allow for high power efficiencies and air stable electrodes, which simplify the encapsulation requirements. While for ferroelectric memristors, they depend on switchable Schottky-to-Ohmic interfacial contacts. Because the ionic polarization related electrochemical doping can modify the interfacial barrier and influence the electric transport property, e.g., changing the contact from Schottky to ohmic-like and vice versa. On the other hand, this polarization as well as the electric modulation can be reversed by the application of an external electric field in the opposite direction, therefore, the doping-induced p-n (or p-i-n) junction is likely to be inverted into a n-p (or n-i-p) junction, resulting in a switchable diode effect in ferroelectric memristors.

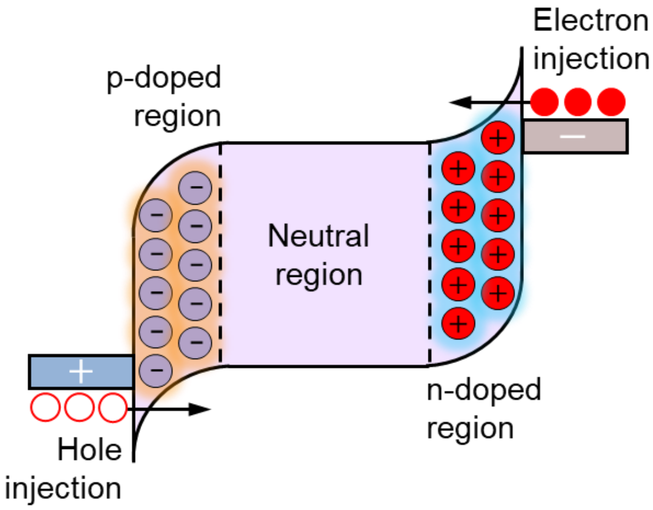

Supplementary Figure 15 | Schematic diagrams of ionic polarization-induced electrochemical doping. After polarization under external electric field (i.e., poling), cations are accumulated in the organic semiconductor near the anode to support p-type doping, resulting in downward band bending and consequent improved hole tunneling (left side in Supplementary Figure 15). At the same time, anions are accumulated in the organic semiconductor near the cathode to support n-type doping, resulting in upward band bending and consequent improved electron tunneling (right side in Supplementary Figure 15). The red round, red circular ring, red round with "+", and purple round with "-" represent electron, hole, cation, and anion, respectively.

Supplementary Note 12. Ion source

SN 12.1 Determining the source of the mobile ions

All the abovementioned phenomena indicate that our memristive switching performance stems from the ion migration-induced barrier modulation rather than the formation/rupture of filaments. Usually, interface-type memristors have been widely reported for perovskite-based materials, in which the mobile ions originate from lattice defects. However, in our work, a series of experiments point out that the mobile ions are provided by the etched PEDOT:PSS:Ag NWs, i.e., they are not from the organic hybrid layer CuPc/C_{60}. As shown in Supplementary Figure 16a and 16b, when the etched PEDOT:PSS:Ag NWs electrode is replaced by an ITO or Ag film, the *I*-*V* hysteresis is negligible. However, when the Ag film electrode is etched for a short time

(~2 s) with the same etching solution, the hysteretic behavior occurs again, but it is very unstable (Supplementary Figure 16c). This result directly implies that the etched Ag film can provide ions but cannot control the ions as effectively as etched PEDOT:PSS:Ag NWs during the repeated sweeping. Moreover, when CuPc is replaced by H_2PC, a similar *I-V* hysteresis is also observed (Supplementary Figure 16d), which not only indicates that the coordinated metallic ions Cu(II) in CuPc do not contribute to the hysteretic phenomenon but also demonstrates that the etched PEDOT:PSS:Ag NWs electrode indeed matters. We also prepared PPA60-based devices with a single CuPc or C_{60} layer as the active material (Supplementary Figure 16e and 16f), and find that both PPA60/CuPc(3 nm)/Au and PPA60/C_{60}(3 nm)/Au exhibit *I-V* hysteresis. However, the hysteresis loops are much weaker than that of PPA60/CuPc(3 nm)/C_{60}(3 nm)/Au. This observation emphasizes that the ultrathin CuPc/C_{60} hybrid layer is very important to achieve excellent resistance switching performance, and also implies that the inserted molecules are not the core factor causing the hysteresis, but instead the etched PEDOT:PSS:Ag NWs electrode.

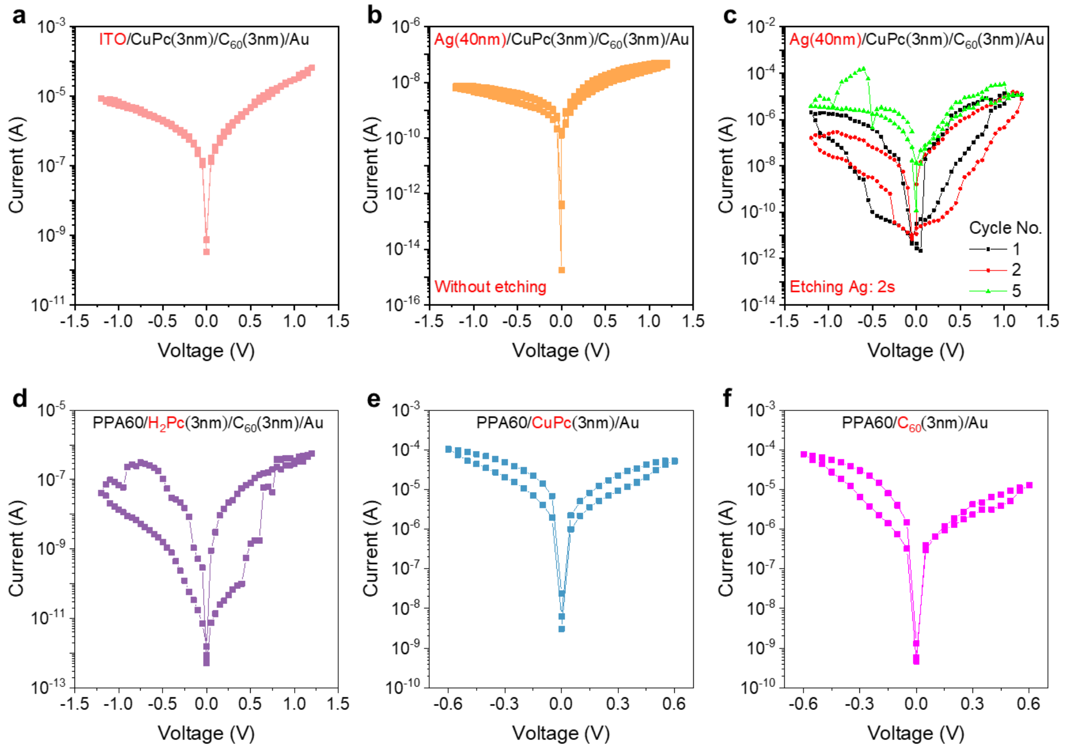

Supplementary Figure 16 | Determining the origin of the mobile ions. a, *I-V* characteristics of the molecular device based on ITO/CuPc (3 nm)/C_{60} (3 nm)/Au tube. **b** and **c**, *I-V*

characteristics of the molecular devices based on Ag (40 nm)/CuPc (3 nm)/C_{60} (3 nm)/Au tube without etching the Ag film (**b**) and with etching the Ag film for a short time (**c**). **d-f**, *I-V* characteristics of the molecular devices based on PPA60/H_2Pc (3 nm)/C_{60} (3 nm)/Au tube (**d**), PPA60/CuPc (3 nm)/Au tube (**e**), and PPA60/C_{60} (3 nm)/Au tube (**f**), respectively.

SN 12.2 Transition between photomultiplication photodiodes and bipolar memristors

Another experimental evidence is that when increasing the water washing time (from 10 s to 10 min) of PPA60 after wet etching (i.e., before molecule deposition), the corresponding molecular devices act as photomultiplication photodiodes again, and the hysteresis behavior is suppressed (Supplementary Figure 17). This is because the longer washing procedure partly removes the mobile ions, leading to a decrease in ion concentration and a reduction of the PPA60/CuPc barrier height, both of which make ion transport difficult. This phenomenon further supports the fact that the etched PEDOT:PSS:AgNWs film is the source of mobile ions. In fact, PEDOT:PSS has been extensively used as an "ion reservoir" to store ions. We use the Raman spectrum and light absorption coefficient profile to characterize the PEDOT:PSS:AgNWs film before and after etching, and find that after etching there are no metallic Ag related signals (see Figure 2h and g in the manuscript). However, the XPS spectrum exhibits a clear Ag signal (see Figure 2j in the manuscript), and the relative atomic content increases with the etching time. If the Ag signal comes from metallic Ag, then the content should decrease as the etching time is prolonged. Obviously, this did not happen. Based on these results, we conclude that Ag nanowires are completely gone after etching and the Ag ions are adsorbed on the etched PEDOT:PSS. Moreover, N is also detected, and the relative atomic content increases with the etching time, which stems from the etching component HNO_3. The mixed etching solution was H_3PO_4 (3):HNO_3 (3):CH_3COOH (23): H_2O (1). In fact, both Ag_3PO_4 and CH_3COOAg exist in solid forms, which may be the reason why element P is not detected.

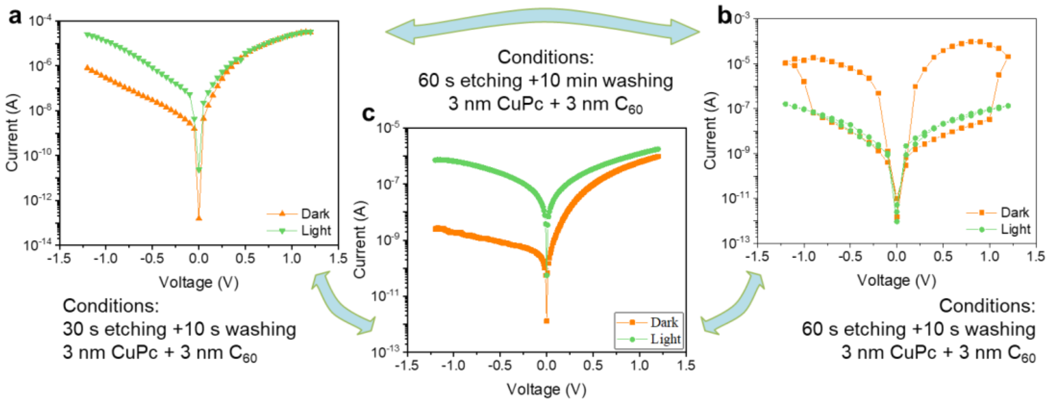

Supplementary Figure 17 | Transition between photomultiplication photodiodes and bipolar memristors. a, *I-V* characteristics of the molecular device based on PEDOT:PSS:AgNWs film wet-etched for 30 s and washed with water for 10 s. **b**, *I-V* characteristics of the molecular device based on PEDOT:PSS:AgNWs film wet-etched for 60 s and washed with water for 10 s. **c**, *I-V* characteristics of molecular device based on PEDOT:PSS:AgNWs film wet-etched for 60 s and washed with water for 10 min.

In summary, we have clarified that our memristive switching performance is attributed to ion migration-induced barrier modulation (instead of the formation/rupture of filaments), and we have provided evidence that the mobile ions originate from the etched PEDOT:PSS film (rather than the organic hybrid layer CuPc/C_{60}).

Supplementary Note 13. Comparison of the memristive performance with previously reported memristors

Here, we focus on bipolar memristors and divide them into two categories according to the sequence of the *I-V* hysteresis loops (Supplementary Figure 12): following the order of (1) HRS→LRS→LRS→HRS (HLLH) or (2) HRS→LRS→HRS→LRS (HLHL), where HRS and LRS are the abbreviations of high resistance state (i.e., OFF state) and low resistance state (i.e., ON state), respectively.

Most HLHL switching phenomena are found in perovskite-based memristors, in which switching is ascribed to the barrier modulation induced by the polarization of the mobile ions in the perovskites or the polarization of the ferroelectric domains. This kind of memristors also

exhibits the “switchable diode effect”. Therefore, they can work as both forward and reverse diodes, which depend on the polarization direction. Such HLHL behaviour and switchable diode effect are rarely reported in molecular/organic memristors but they occur in our devices. Compared with previously reported perovskite-based memristors the performance of our devices is comparable in terms of the rectification ratio, ON/OFF ratio, switching voltage, and retention stability. On the other hand, the ON-state current density of our devices is higher than that of most perovskite-based memristors. Moreover, there is no need to conduct a poling process to activate the memristive switching behaviour in the molecular devices in our work. Poling refers to applying a higher voltage over a period of time to create polarization in perovskite materials. For the molecular devices in our work, it is supposed that the ultrahigh electric field (for example, the average electric field is about 1.67 MV/cm at 1 V) immediately polarizes the mobile ions through the ultrathin molecular hybrid layer. However, compared to the HLLH memristors listed in Supplementary Table 3, the endurance capability of our devices still needs to be enhanced.

Supplementary Table 3 | Comparison of the memristive performance with previously reported memristors

Device structure[a]	Thickness (nm)	Hysteresis order[b]	Forward RR@±V[c]	Reverse RR@±V	ON/OFF ratio@V	Switching voltage (V)	ON-state current density (A/cm^2)	Forming required?[d]	Retention (s)	Endurance (cycles)	Ref.
Ag/SAM/GaO_x/EGaIn	~2	HLLH	–	2.5×10^4 @1.0 V[e]	6.7×10^3 @-0.3 V	-0.89	~10^{-2} @-0.5 V	No	1.2×10^4	10^6	61
ITO/Au NPs/ $[Ru(L)_3](PF_6)_2$/ITO	10-100	HLLH	–	–	~10^5 @0.3 V	~0.52	~10^3 @0.3	No	>10^6	~10^{12}	62
Pt/PA-TsOH/Pt	90	HLLH	–	–	>10^5 @0.1 V	~7.0	–	No	10^5	>10^3	63
ITO/Poly-1^{4+}/Al	100	HLLH	–	–	~2.0×10^2 @1.0 V	3.4	~10^{-2} @1.0	No	1.2×10^4	500	64
Ag/MoS_2/MoO_x/Ag	50-100	HLLH	–	–	~10^6 @~0 V	0.1-0.2	~10 @0.01	No	~10^4	3.0×10^4	65
ITO/$CsPbBr_3$ ODs/PMMA/Ag	~800	HLLH	–	–	~10^3 @0.5 V	~1.0	–	Electro-forming	10^5	10^4	66
Pt/BCZT/HAO/Au	50	HLHL	~10^4 @0.1 V	~10^4 @0.1 V	~10^4 @-0.1 V	~0.2	~10^{-5} @-0.1 V	Not specified	10^4	60	67
FTO/$BiFeO_3$/C-AFM	250	HLHL	~10^4 @2.0 V	~7 @4.0 V	~7.0×10^3 @2.0 V	~1.0	~10^{-2} @2.0 V	Poling @±9V	10^3	–	68
STO/$BiFeO_3$/C-AFM	240	HLHL	–	~100 @1.0 V	~10^3 @-2.0 V	~-1.0	~10^{-2} @-2.0 V	Poling @±10V	10^6	–	49
STO/SRO/$BiFeO_3$/ C-AFM	40	HLHL	~500 @0.6 V	~200 @0.6 V	~753 @0.5 V	~2.0	–	Poling @±3.5V	10^3	–	41

(Continued Supplementary Table 3)

Device structure[a]	Thickness (nm)	Hysteresis order[b]	Forward RR@±V[c]	Reverse RR@±V	ON/OFF ratio@V	Switching voltage (V)	ON-state current density (A/cm^2)	Forming required?[d]	Retention (s)	Endurance (cycles)	Ref.
ITO/PEDOT:PSS/ $MAPbI_3$/Au	300	HLHL	~200 @1.0 V	~300 @1.0 V	>10^3 @-1.0 V	~-1.0	~10^{-2} @-1.0 V	Poling @±2.5V	–	–	43
SRO/$BiFeO_3$(Ca)/ C-AFM	100	HLHL	–	~10^3 @10 V	~10^3 @-2.0 V	~-5.0	–	Poling @±12V	–	–	44
PPA60/CuPc/C_{60}/Au	6	HLHL	~400 @0.5 V	~900 @0.5 V	~3×10^3 @0.5 V	~0.8	>10 @0.5 V	No	2×10^3	100	This work

a, The Red part indicates the storage medium.

b, According to the direction of the *I-V* hysteresis loops, bipolar memristors are simply divided into two categories: following the order of (1) HRS→LRS→LRS→HRS (HLLH) or (2) HRS→LRS→HRS→LRS (HLHL), where HRS and LRS are the abbreviations of high resistance state (i.e., OFF state) and low resistance state (i.e., ON state), respectively.

c, RR= Rectification Ratio.

d, The "forming" in filamentary memristors refers to "electroforming", while in ferroelectric-type memristors it means "poling".

e, In this work, the rectifying behavior arises from the extremely asymmetrical *I-V* hysteresis loops, rather than the "switchable diode effect" that usually occurs in ferroelectric-type memristors.

Supplementary References

1. Haick, H., Cahen, D. Making contact: Connecting molecules electrically to the macroscopic world. *Prog. Surf. Sci.* **83**, 217-261 (2008).

2. Liu, Y., Qiu, X., Soni, S., Chiechi, R.C. Charge transport through molecular ensembles: Recent progress in molecular electronics. *Chem. Phys. Rev.* **2**, 021303 (2021).

3. Gabler, F., Karnaushenko, D.D., Karnaushenko, D., Schmidt, O.G. Magnetic origami creates high performance micro devices. *Nat. Commun.* **10**, 1-10 (2019).

4. Akbar, F., Rivkin, B., Aziz, A., Becker, C., Karnaushenko, D.D., Medina-Sánchez, M., Karnaushenko, D., Schmidt, O.G. Self-sufficient self-oscillating microsystem driven by low power at low Reynolds numbers. *Sci. Adv.* **7**, eabj0767 (2021).

5. Yin, Y., Wang, J., Wang, X., Li, S., Jorgensen, M.R., Ren, J., Meng, S., Ma, L., Schmidt, O.G. Water nanostructure formation on oxide probed in situ by optical resonances. *Sci. Adv.* **5**, eaax6973 (2019).

6. Bandari, V.K., Nan, Y., Karnaushenko, D., Hong, Y., Sun, B., Striggow, F., Karnaushenko, D.D., Becker, C., Faghih, M., Medina-Sánchez, M. A flexible microsystem capable of controlled motion and actuation by wireless power transfer. *Nat. Electron.* **3**, 172-180 (2020).

7. Lee, Y., Bandari, V.K., Li, Z., Medina-Sánchez, M., Maitz, M.F., Karnaushenko, D., Tsurkan, M.V., Karnaushenko, D.D., Schmidt, O.G. Nano-biosupercapacitors enable autarkic sensor operation in blood. *Nat. Commun.* **12**, 1-10 (2021).

8. Li, T., Bandari, V.K., Hantusch, M., Xin, J., Kuhrt, R., Ravishankar, R., Xu, L., Zhang, J., Knupfer, M., Zhu, F., Yan, D., Schmidt, O.G. Integrated molecular diode as 10 MHz half-wave rectifier based on an organic nanostructure heterojunction. *Nat. Commun.* **11**, 3592 (2020).

9. Pourhossein, P., Chiechi, R.C. Directly addressable sub-3 nm gold nanogaps fabricated by nanoskiving using self-assembled monolayers as templates. *ACS Nano* **6**, 5566-5573 (2012).

10. Bonifas, A.P., McCreery, R.L. 'Soft' Au, Pt and Cu contacts for molecular junctions through surface-diffusion-mediated deposition. *Nat. Nanotechnol.* **5**, 612-617 (2010).

11. Karuppannan, S.K., Neoh, E.H.L., Vilan, A., Nijhuis, C.A. Protective layers based on carbon paint to yield high-quality large-area molecular junctions with low contact resistance. *J. Am. Chem. Soc.* **142**, 3513-3524 (2020).

12. Hnid, I., Frath, D., Lafolet, F., Sun, X., Lacroix, J.-C. Highly efficient photoswitch in diarylethene-based molecular junctions. *J. Am. Chem. Soc.* **142**, 7732-7736 (2020).

13. Seferos, D.S., Trammell, S.A., Bazan, G.C., Kushmerick, J.G. Probing π-coupling in molecular junctions. *Proc. Natl Acad. Sci. USA* **102**, 8821-8825 (2005).

14. Nijhuis, C.A., Reus, W.F., Barber, J.R., Dickey, M.D., Whitesides, G.M. Charge transport and rectification in arrays of SAM-based tunneling junctions. *Nano Lett.* **10**, 3611-3619 (2010).

15. Chiechi, R.C., Weiss, E.A., Dickey, M.D., Whitesides, G.M. Eutectic gallium–indium (EGaIn): a moldable liquid metal for electrical characterization of self-assembled monolayers. *Angew. Chem. Int. Ed.* **47**, 142-144 (2008).

16. Kayser, B., Fereiro, J.A., Bhattacharyya, R., Cohen, S.R., Vilan, A., Pecht, I., Sheves, M., Cahen, D. Solid-state electron transport via the protein azurin is temperature-independent down to 4 K. *J. Phys. Chem. Lett.* **11**, 144-151 (2019).

17. Yoon, H.P., Maitani, M.M., Cabarcos, O.M., Cai, L., Mayer, T.S., Allara, D.L. Crossed-nanowire molecular junctions: a new multispectroscopy platform for conduction--structure correlations. *Nano Lett.* **10**, 2897-2902 (2010).

18. Tefashe, U.M., Nguyen, Q.V., Lafolet, F., Lacroix, J.-C., McCreery, R.L. Robust Bipolar Light Emission and Charge Transport in Symmetric Molecular Junctions. *J. Am. Chem. Soc.* **139**, 7436-7439 (2017).

19. Bergren, A.J., Zeer-Wanklyn, L., Semple, M., Pekas, N., Szeto, B., McCreery, R.L. Musical molecules: the molecular junction as an active component in audio distortion circuits. *J. Phys. Condens. Matter* **28**, 094011 (2016).

20. Pourhossein, P., Vijayaraghavan, R.K., Meskers, S.C., Chiechi, R.C. Optical modulation of nano-gap tunnelling junctions comprising self-assembled monolayers of hemicyanine dyes. *Nat. Commun.* **7**, 1-9 (2016).

21. Wang, W., Lee, T., Reed, M.A. Mechanism of electron conduction in self-assembled alkanethiol monolayer devices. *Phys. Rev. B* **68**, 035416 (2003).

22. Akkerman, H.B., Blom, P.W., de Leeuw, D.M., de Boer, B. Towards molecular electronics with large-area molecular junctions. *Nature* **441**, 69-72 (2006).

23. Vilan, A., Cahen, D. Soft contact deposition onto molecularly modified GaAs. Thin metal film flotation: principles and electrical effects. *Adv. Funct. Mater.* **12**, 795-807 (2002).

24. Loo, Y.-L., Lang, D.V., Rogers, J.A., Hsu, J.W. Electrical contacts to molecular layers by nanotransfer printing. *Nano Lett.* **3**, 913-917 (2003).

25. Wang, Z., Dong, H., Li, T., Hviid, R., Zou, Y., Wei, Z., Fu, X., Wang, E., Zhen, Y., Nørgaard, K. Role of redox centre in charge transport investigated by novel self-assembled conjugated polymer molecular junctions. *Nat. Commun.* **6**, 1-10 (2015).

26. Spyropoulos, G.D., Gelinas, J.N., Khodagholy, D. Internal ion-gated organic electrochemical transistor: A building block for integrated bioelectronics. *Sci. Adv.* **5**, eaau7378 (2019).

27. Tybrandt, K., Zozoulenko, I.V., Berggren, M. Chemical potential-electric double layer coupling in conjugated polymer-polyelectrolyte blends. *Sci. Adv.* **3**, eaao3659 (2017).

28. Jansen-van Vuuren, R.D., Armin, A., Pandey, A.K., Burn, P.L., Meredith, P. Organic photodiodes: the future of full color detection and image sensing. *Adv. Mater.* **28**, 4766-4802 (2016).

29. Clifford, J.P., Konstantatos, G., Johnston, K.W., Hoogland, S., Levina, L., Sargent, E.H. Fast, sensitive and spectrally tuneable colloidal-quantum-dot photodetectors. *Nat. Nanotechnol.* **4**, 40-44 (2009).

30. Guo, F., Yang, B., Yuan, Y., Xiao, Z., Dong, Q., Bi, Y., Huang, J. A nanocomposite ultraviolet photodetector based on interfacial trap-controlled charge injection. *Nat. Nanotechnol.* **7**, 798-802 (2012).

31. Wang, J., Han, J., Chen, X., Wang, X. Design strategies for two-dimensional material photodetectors to enhance device performance. *InfoMat* **1**, 33-53 (2019).

32. Miao, J., Zhang, F. Recent Progress on Photomultiplication Type Organic Photodetectors. *Laser Photonics Rev.* **13**, 1800204 (2018).

33. Hammond, W. T. & Xue, J. Organic heterojunction photodiodes exhibiting low voltage, imaging-speed photocurrent gain. *Appl. Phys. Lett.* **97**, 073302 (2010).

34. Li, L., Zhang, F., Wang, W., An, Q., Wang, J., Sun, Q., Zhang, M. Trap-assisted photomultiplication polymer photodetectors obtaining an external quantum efficiency of 37,500%. *ACS Appl. Mater. Interfaces* **7**, 5890-5897 (2015).

35. Jang, M.S., Yoon, S., Sim, K.M., Cho, J., Chung, D.S. Spatial Confinement of the Optical Sensitizer to Realize a Thin Film Organic Photodetector with High Detectivity and Thermal Stability. *J. Phys. Chem. Lett.* **9**, 8-12 (2018).

36. Xiang, D., Wang, X., Jia, C., Lee, T., Guo, X. Molecular-Scale Electronics: From Concept to Function. *Chem. Rev.* **116**, 4318-4440 (2016).

37. Song, H., Kim, Y., Jang, Y.H., Jeong, H., Reed, M.A., Lee, T. Observation of molecular orbital gating. *Nature* **462**, 1039-1043 (2009).

38. Choi, S.H., Kim, B., Frisbie, C.D. Electrical resistance of long conjugated molecular wires. *Science* **320**, 1482-1486 (2008).

39. Lee, B.-H., Bae, H., Seong, H., Lee, D.-I., Park, H., Choi, Y.J., Im, S.-G., Kim, S.O., Choi, Y.-K. Direct observation of a carbon filament in water-resistant organic memory. *ACS Nano* **9**, 7306-7313 (2015).

70. Raeis Hosseini, N., Lee, J.-S. Resistive switching memory based on bioinspired natural solid polymer electrolytes. *ACS Nano* **9**, 419-426 (2015).

41. Hong, S., Choi, T., Jeon, J.H., Kim, Y., Lee, H., Joo, H.Y., Hwang, I., Kim, J.S., Kang, S.O., Kalinin, S.V. Large resistive switching in ferroelectric $BiFeO_3$ nano-island based switchable diodes. *Adv. Mater.* **25**, 2339-2343 (2013).

42. Chen, A., Zhang, W., Dedon, L.R., Chen, D., Khatkhatay, F., MacManus-Driscoll, J.L., Wang, H., Yarotski, D., Chen, J., Gao, X., Martin, L.W., Roelofs, A., Jia, Q. Couplings of Polarization with Interfacial Deep Trap and Schottky Interface Controlled Ferroelectric Memristive Switching. *Adv. Funct. Mater.* **30**, 2000664 (2020).

43. Xiao, Z., Yuan, Y., Shao, Y., Wang, Q., Dong, Q., Bi, C., Sharma, P., Gruverman, A., Huang, J. Giant switchable photovoltaic effect in organometal trihalide perovskite devices. *Nat. Mater.* **14**, 193-198 (2015).

44. Yang, C.H., Seidel, J., Kim, S.Y., Rossen, P.B., Yu, P., Gajek, M., Chu, Y.H., Martin, L.W., Holcomb, M.B., He, Q., Maksymovych, P., Balke, N., Kalinin, S.V., Baddorf, A.P., Basu, S.R., Scullin, M.L., Ramesh, R. Electric modulation of conduction in multiferroic Ca-doped BiFeO3 films. *Nat. Mater.* **8**, 485-493 (2009).

45. Sajedi Alvar, M., Blom, P.W.M., Wetzelaer, G.A.H. Space-charge-limited electron and hole currents in hybrid organic-inorganic perovskites. *Nat. Commun.* **11**, 4023 (2020).

46. Lim, E., Ismail, R. Conduction Mechanism of Valence Change Resistive Switching Memory: A Survey. *Electronics* **4**, 586-613 (2015).

47. Liu, L., Li, Y., Huang, X., Chen, J., Yang, Z., Xue, K.H., Xu, M., Chen, H., Zhou, P., Miao, X. Low-Power Memristive Logic Device Enabled by Controllable Oxidation of 2D $HfSe_2$ for In-Memory Computing. *Adv. Sci.* **8**, 2005038 (2021).

48. Li, Y., Zhou, Y.-X., Xu, L., Lu, K., Wang, Z.-R., Duan, N., Jiang, L., Cheng, L., Chang, T.-C., Chang, K.-C. Realization of functional complete stateful Boolean logic in memristive crossbar. *ACS Appl. Mater. Interfaces* **8**, 34559-34567 (2016).

49. Jiang, A.Q., Wang, C., Jin, K.J., Liu, X.B., Scott, J.F., Hwang, C.S., Tang, T.A., Lu, H.B., Yang, G.Z. A resistive memory in semiconducting $BiFeO_3$ thin-film capacitors. *Adv. Mater.* **23**, 1277-1281 (2011).

50. Lee, J.H., Jeon, J.H., Yoon, C., Lee, S., Kim, Y.S., Oh, T.J., Kim, Y.H., Park, J., Song, T.K., Park, B.H. Intrinsic defect-mediated conduction and resistive switching in multiferroic $BiFeO_3$ thin films epitaxially grown on $SrRuO_3$ bottom electrodes. *Appl. Phys. Lett.* **108**, 112902 (2016).

51. Choi, T., Lee, S., Choi, Y.J., Kiryukhin, V., Cheong, S.W. Switchable ferroelectric diode and photovoltaic effect in BiFeO3. *Science* **324**, 63-66 (2009).

52. Kwon, K.C., Hong, K., Van Le, Q., Lee, S.Y., Choi, J., Kim, K.B., Kim, S.Y., Jang, H.W. Inhibition of ion migration for reliable operation of organolead halide perovskite-based Metal/Semiconductor/Metal broadband photodetectors. *Adv. Funct. Mater.* **26**, 4213-4222 (2016).

53. Lan, D. The physics of ion migration in perovskite solar cells: Insights into hysteresis, device performance, and characterization. *Prog. Photovolt. Res. Appl.* **28**, 533-537 (2020).

54. Lan, C., Zou, H., Wang, L., Zhang, M., Pan, S., Ma, Y., Qiu, Y., Wang, Z.L., Lin, Z. Revealing Electrical-Poling-Induced Polarization Potential in Hybrid Perovskite Photodetectors. *Adv. Mater.* **32**, 2005481 (2020).

55. Meier, S.B., Tordera, D., Pertegás, A., Roldán-Carmona, C., Ortí, E., Bolink, H.J. Light-emitting electrochemical cells: recent progress and future prospects. *Mater. Today* **17**, 217-223 (2014).

56. Asadi, K., de Leeuw, D.M., de Boer, B., Blom, P.W. Organic non-volatile memories from ferroelectric phase-separated blends. *Nat. Mater.* **7**, 547-550 (2008).

57. Sangwan, V.K., Lee, H.S., Bergeron, H., Balla, I., Beck, M.E., Chen, K.S., Hersam, M.C. Multi-terminal memtransistors from polycrystalline monolayer molybdenum disulfide. *Nature* **554**, 500-504 (2018).

58. O'Kane, S.E.J., Richardson, G., Pockett, A., Niemann, R.G., Cave, J.M., Sakai, N., Eperon, G.E., Snaith, H.J., Foster, J.M., Cameron, P.J., Walker, A.B. Measurement and modelling of dark current decay transients in perovskite solar cells. *J. Mater. Chem. C* **5**, 452-462 (2017).

59. Khan, Y., Ahn, Y., Seo, J.H., Walker, B. Ionic moieties in organic and hybrid semiconducting devices: influence on energy band structures and functions. *J. Mater. Chem. C* **8**, 13953-13971 (2020).

60. Matyba, P., Maturova, K., Kemerink, M., Robinson, N.D., Edman, L. The dynamic organic p-n junction. *Nat. Mater.* **8**, 672-676 (2009).

61. Han, Y., Nickle, C., Zhang, Z., Astier, H., Duffin, T.J., Qi, D., Wang, Z., Del Barco, E., Thompson, D., Nijhuis, C.A. Electric-field-driven dual-functional molecular switches in tunnel junctions. *Nat. Mater.* **19**, 843-848 (2020).

62. Goswami, S., Matula, A.J., Rath, S.P., Hedstrom, S., Saha, S., Annamalai, M., Sengupta, D., Patra, A., Ghosh, S., Jani, H., Sarkar, S., Motapothula, M.R., Nijhuis, C.A., Martin, J., Goswami, S., Batista, V.S., Venkatesan, T. Robust resistive memory devices using solution-processable metal-coordinated azo aromatics. *Nat. Mater.* **16**, 1216-1224 (2017).

63. Hu, B., Zhu, X., Chen, X., Pan, L., Peng, S., Wu, Y., Shang, J., Liu, G., Yan, Q., Li, R.W. A multilevel memory based on proton-doped polyazomethine with an excellent uniformity in resistive switching. *J. Am. Chem. Soc.* **134**, 17408-17411 (2012).

64. Cui, B.-B., Mao, Z., Chen, Y., Zhong, Y.-W., Yu, G., Zhan, C., Yao, J. Tuning of resistive memory switching in electropolymerized metallopolymeric films. *Chem. Sci.* **6**, 1308-1315 (2015).

65. Bessonov, A.A., Kirikova, M.N., Petukhov, D.I., Allen, M., Ryhanen, T., Bailey, M.J. Layered memristive and memcapacitive switches for printable electronics. *Nat. Mater.* **14**, 199-204 (2015).

66. Yen, M.C., Lee, C.J., Liu, K.H., Peng, Y., Leng, J., Chang, T.H., Chang, C.C., Tamada, K., Lee, Y.J. All-inorganic perovskite quantum dot light-emitting memories. *Nat. Commun.* **12**, 4460 (2021).

67. Silva, J., Silva, J.P.B., Sekhar, K., Pereira, M., Gomes, M. Impact of the ferroelectric layer thickness on the resistive switching characteristics of ferroelectric/dielectric structures. *Appl. Phys. Lett.* **113**, 102904 (2018).

68. Zhang, L., Chen, J., Cao, J., He, D., Xing, X. Large resistive switching and switchable photovoltaic response in ferroelectric doped $BiFeO_3$-based thin films by chemical solution deposition. *J. Mater. Chem. C* **3**, 4706-4712 (2015).
